# A Real-Time Fire Detection Method from Video with Multifeature Fusion

**DOI:** 10.1155/2019/1939171

**Published:** 2019-07-14

**Authors:** Faming Gong, Chuantao Li, Wenjuan Gong, Xin Li, Xiangbing Yuan, Yuhui Ma, Tao Song

**Affiliations:** ^1^Department of Computer and Communication Engineering, China University of Petroleum, Qingdao 266580, China; ^2^China Petroleum and Chemical Corporation Shengli Oilfield Branch Ocean Oil Production Plant, Dongying, Shandong, China; ^3^Department of Artificial Intelligence, Faculty of Computer Science, Polytechnical University of Madrid, Campus de Montegancedo, Boadilla del Monte, 28660 Madrid, Spain

## Abstract

The threat to people's lives and property posed by fires has become increasingly serious. To address the problem of a high false alarm rate in traditional fire detection, an innovative detection method based on multifeature fusion of flame is proposed. First, we combined the motion detection and color detection of the flame as the fire preprocessing stage. This method saves a lot of computation time in screening the fire candidate pixels. Second, although the flame is irregular, it has a certain similarity in the sequence of the image. According to this feature, a novel algorithm of flame centroid stabilization based on spatiotemporal relation is proposed, and we calculated the centroid of the flame region of each frame of the image and added the temporal information to obtain the spatiotemporal information of the flame centroid. Then, we extracted features including spatial variability, shape variability, and area variability of the flame to improve the accuracy of recognition. Finally, we used support vector machine for training, completed the analysis of candidate fire images, and achieved automatic fire monitoring. Experimental results showed that the proposed method could improve the accuracy and reduce the false alarm rate compared with a state-of-the-art technique. The method can be applied to real-time camera monitoring systems, such as home security, forest fire alarms, and commercial monitoring.

## 1. Introduction

With the rapid spread of urbanization in the world, both the number of permanent residents in cities and the population density are increasing. When a fire occurs, it seriously threatens people's lives and causes major economic losses. According to incomplete statistics, there were 312,000 fires in the country in 2016, with 1,582 people killed and 1,065 injured, and a direct property loss of 3.72 billion dollars [1, 2]. In March and April of 2019, there were many large-scale fire accidents around the world, such as forest fires in Liangshan, China, the Notre Dame fire in France, forest fires in Italy, and the grassland fire in Russia, which caused great damage to people's lives and property. Therefore, fire detection is vitally important to protecting people's lives and property. The current detection methods in cities rely on various sensors for detection [3–6], including smoke alarms, temperature alarms, and infrared ray alarms. Although these alarms can play a role, they have major flaws. First, a certain concentration of particles in the air must be reached to trigger an alarm. When an alarm is triggered, a fire may already be too strong to control, defeating the purpose of early warning. Second, most of the alarms can only be functional in a closed environment, which is ineffective for a wide space, such as outdoors or public spaces. Third, there may be false alarms. When the nonfire particle concentration reaches the alarm concentration, it will automatically sound the alarm. Human beings cannot intervene and get the latest information in time.

To prevent fires and hinder their rapid growth, it is necessary to establish a monitoring system that can detect early fires. Establishing a camera-based automatic fire monitoring algorithm can achieve 24/7 automatic monitoring without interruption, which greatly reduces labor costs, and the rapid spread of urban monitoring systems provides the groundwork for camera-based fire detection [7]. Greatly reducing the cost increases the economic feasibility of such systems.

This paper proposed a fire detection method based on the multifeatures of flame, which firstly combined frame difference detection [8] screening nonmoving fire pixels with RGB color model [9] screening nonfire color pixels. In the preprocessing module, the frame difference detection operates quickly and does not include complex calculations, has low environmental requirements, and does not need to consider the time of day, weather, and other factors. In addition, the adopted RGB/HIS color model is relatively stable. Then, taking into the account the spatial variability, the area variability, boundary complexity, and shape variability properties, the characteristics of the flame are determined by using the number of flame pixel points, the convex hull [10], and the centroid. Finally, a mature support vector machine is used for verification.

The camera-based fire monitoring system can monitor the specified area in real time through video processing. When a fire is detected based on the video, it will send a captured alarm image to the administrator. The administrator makes a final confirmation based on the submitted alarm image. For example, when an accident occurs on a highway and causes a fire, based on the image transmitted by the detection algorithm, one can immediately rescue the victims, saving precious time and minimizing damage.

The main contributions of this paper are as follows: (1) We combine motion detection based on frame difference with color detection based on the RGB/HSI model. Color detection is only for regions of motion that the motion detection phase is completed. Our method has improved the precision and reduced redundant calculation. In addition, we have improved the frame difference method. (2) According to the spatial correlation between consecutive image frames, we have improved the traditional methods of detecting fire from one single image frame. Temporal information is combined with the flame features through a space-time flame centroid stability-based detection method. At the same time, we combine the data obtained during the fire preprocessing phase to reduce computational redundancy and computational complexity. (3) We extracted various flame features, spatial variability, shape variability, and area variability. We used the support vector machine to train, complete the final verification, reduce the false negatives rate and false positives rate, and improve the accuracy.

## 2. Related Works

For fire detection, the traditional method is to use a sensor for detection. One of the defects is the high false rate because the trigger alarm is based on the concentration of particles or the surrounding temperature and is therefore easily disturbed by the surroundings. At the same time, this method cannot know the location and the real-time status of the fire. For outdoor scenes, which are notable fire hazards, this type of sensor cannot provide effective detection. Due to the many problems in traditional fire identification, how to accurately identify the fire has received great attention. Therefore, fire detection has achieved rapid development in the direction of fire detection sensors, improvement in detection equipment, and fire detection based on video images.

Khan et al. [11] proposed a method based on video using flame dynamics and static indoor flame detection, using the color, perimeter, area, and roundness of the flame. Their method takes a small fire such as a candle as an unimportant part. By removing and then applying the flame growth characteristics to judge, this method may have a big problem in early fire warning. Seebamrungsat et al. [12] proposed a rule based on the combination of HSV and YCbCr. Their system requires extra conversion of color space and is therefore better than using only one color space method, but their work only uses the static characteristics of the flame. The method is relatively fragile and not stable enough. Foggia et al. [13] proposed a novel motion detection method based on the disordered nature of the flame-word bag strategy. Chen and Huang [14] proposed a Gaussian model to simulate HSV and analyze the time and space factors of the flame, but the Gaussian mixture model requires higher calculation time and the analysis is fuzzier. Krüger et al. [6] used a hydrogen sensor. The above method improves the traditional fire detection sensors, which improve the reliability of the detection and reduce the detection sensitivity. However, the use of the sensor has limitations due to the different properties of the combustion product. Burnett and Wing [15] used a new low-cost camera that can reduce the interference of smoke on the flame and has excellent detection capabilities for RGB and HSV, but there are still some limitations in the popularity and application of this camera. Töreyin et al. [16] proposed a Gaussian mixture background estimation method for detecting motion pixels in video. This method selects candidate fire regions by color model and then performs wavelet analysis in time and space domains to determine high frequency activity in the region. Similar to the previous problem, this method has high computational complexity in practical applications. Han et al. [17] used motion detection based on the Gaussian model and multicolor model and obtained good experimental results. However, since Gaussian models and color models require a large amount of computational time, they cannot be applied to actual scenes. Chen et al. [18] improved the traditional flame detection method, and the flame flickering detection algorithm is incorporated into the scheme to detect fires in color video sequences. Testing results show that the proposed algorithms are effective, robust, and efficient. However, the calculation speed is slow, suitable for 320 ∗ 240 images. It may not be suitable for high quality images. Dimitropoulos et al. and Çetin et al. [19, 20] used a variety of flame characteristics to judge and achieved good results. Hashemzadeh and Zademehdi [21] proposed a candidate fire pixel detection technique based on ICA K-medoids, which is the basis for practical applications. Savcı et al. [22] proposed a new method based on Markov002E. Kim et al. [23] used an ultraspectral camera to overcome the limitations of RGB cameras which cannot distinguish between flame and general light source (halogen, LED). According to the experimental results, they have achieved good results, and there may be limitations, such as higher camera costs. Wu et al. [24] proposed based on the combination of radiation domain feature models. Giwa and Benkrid [25] proposed a new color-differentiating conversion matrix that is robust against false alarm. Patel et al. [26] proposed technique has been using the color cue and flame flicker for detecting fire. At present, deep learning has become an active topic due to its high accuracy of recognition in a wide range of applications. In the studies [27–29], a deep learning method for fire detection is used and high accuracy is achieved. We can use deep learning technology to solve the problems we have in the process of flame detection. However, there are certain limitations. For example, deep learning can have better accuracy under big data, but the number of fires is less, and the actual flame samples taken by using the camera are less. Deep learning requires higher performance equipment and training that takes more time. For example, in the study by Alves et al. [30], the flame dataset is 800 images.

From what has been discussed above, fire detection based on video has rapidly been studied and developed with multifield technology used to solve the existing limitation of current methods, but there are still some problems. Compared with the images in the experiment, the camera images may not have rich color information and therefore may cause a higher false negative rate. If the algorithm involves fewer flame characteristics, higher false positives may occur. Taking practicality into account, traditional fire detection needs to be optimized.

**Figure 1:**
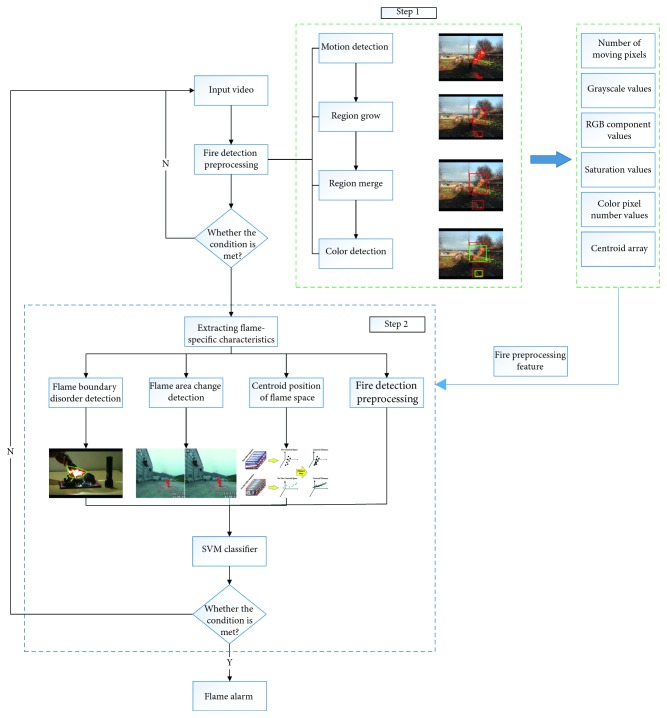
Flowchart of the algorithm. In the judgment statement after preprocessing, condition 1 indicates whether the object to be detected satisfies the requirements, that is, the basic features (color and dynamics) of the flame. In the judgment statement of step 2, condition 2 indicates whether the extracted feature vector is the correct category after calculation.

In this paper, fire preprocessing methods are presented in Section 3, including motion detection and color detection. The extraction method of the advanced features of the flame and the classification method based on the support vector machine are presented in Section 4. The experimental results are presented in Section 5. Finally, conclusions are drawn in the last section. The algorithm flowchart is shown in Figure 1.

## 3. Fire Detection Preprocessing

### 3.1. Flame Features

We extracted several flame characteristics. For static features, color is one of the most obvious feature of flames, which are usually red. In general, using color profiles is an effective method for fire detection. As for dynamic features, the flame has abundant characteristics. First, the flame has no fixed shape. Second, there is disorder in the boundaries of the flame. Third, the fire location has a certain similarity in the continuous image. Based on the analysis of flame features, we have designed fire detection module that takes into account multifeatures of the flame.

### 3.2. Motion Detection Based on Improved Frame Difference Method

Due to the dynamic nature of fire, the shape of the flame is irregular and changes constantly. So when using fire as a significant feature for motion detection, the usual detection methods are continuous frame changes [31], background subtraction [32], and mixed Gaussian background modeling [33]. The background subtraction needs to properly set the background because there is a large gap between day and night; in general, it is hard to have a constant, and one must set parameters for it, which is more complicated than a static background. The mixed Gaussian model is too complex and needs to set the history frame, Gaussian mixture number, background update rate, and noise at the preprocessing stage. Therefore, this algorithm is not suitable for preprocessing. The advantage of the frame difference method is simple to implement, the programming complexity is low, and it is not sensitive to scene changes such as light and can adapt to various dynamic environments with good stability. The disadvantage is that the complete area of the object cannot be extracted. There is an “empty hole” inside the object, and only the boundary can be extracted. Therefore, an improved frame difference method is adopted in this paper.

**Figure 2:**
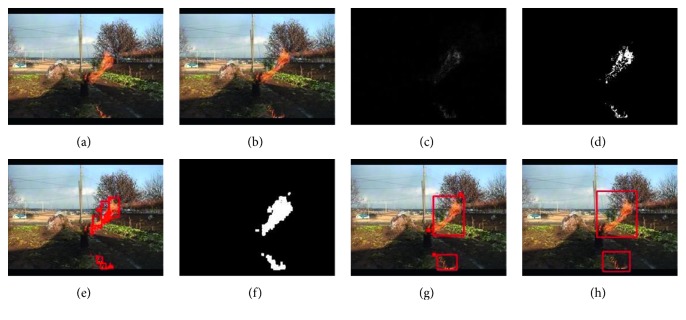
Motion detection based on the improved frame difference method. (a) The previous frame image. (b) The current frame image. (c) The frame difference result image. (d) The resulting image of the thresholding of (c). (e) A diagram showing the result of the region of the moving pixel in (d). It can be seen from (e) that if the optimization process is not performed, directly performing color detection on the rectangle in the image will increase the complexity of the algorithm, and it is not conducive to the use of preprocessed data when extracting advanced features later. (f) The binary image after the expansion of (d). (g) The region image of the moving pixels of (f). Regions of similar motion pixels in (f) are merged. (h) The region diagram of its motion pixels.

Since the airflow and the properties of the combustion itself will cause a constant change in the flame pixels [34], nonfire pixel images can be removed by comparing two consecutive images. We use an improved frame difference algorithm. First, the video stream is converted into a frame image. Second, the image is processed in grayscale to convert three RGB channels into a single channel, which saves calculation time. Third, if the image is an initial frame, an initialization operation is performed. For the other frame images, the frame difference with the previous frame is used. The frame difference formula is shown in (1). Then, denoising operations are performed, and the denoising formula is as shown in (2). Fourth, the denoised image is dilated to enhance the connectivity of the region and reduce the computational complexity in the color detection phase. The formula for the dilation operation is as shown in (3). Fifth, regional optimization is done. Contour detection is performed on the dilated image. If the condition is not satisfied, the frame is removed, and the next frame of image is used for detection. Regions are merged based on the distance between rectangles. Finally, the merged coordinates of the region are passed to the flame color detection phase:

(1) $$\begin{array}{c} F_{\text{v}}\left(x,y\right)=\left|F_{\text{c}}\left(x,y\right)-F_{\text{p}}\left(x,y\right)\;\;\right|, \end{array}$$

where *F*_c_(*x*, *y*) is the current frame, as shown in Figure 2(b). *F*_p_(*x*, *y*) is the previous frame, as shown in Figure 2(a). *F*_v_(*x*, *y*) is the difference between the current frame and the previous frame, as shown in Figure 2(c). We cannot directly use the image after the frame difference; we need to denoise and use the threshold to segment out the parts we need:

(2) $$\begin{array}{c} F_{t}\left(x,y\right)=\left\{\begin{array}{cc} \text{maxval,} & \text{if }F_{\text{v}}\left(x,y\right)>\text{thresh,}\\ 0, & \text{otherwise,} \end{array}\right\}, \end{array}$$

where *F*_v_(*x*, *y*) is the difference between the current frame and the previous frame, which is assigned to the max value if its pixel is greater than the set threshold, otherwise set to zero. Thresh is the set threshold, and here we set 20 to ensure that the motion pixels are not removed. *F*_*t*_(*x*, *y*) is the result of denoising, as shown in Figure 2(d). It can be seen from Figure 2(e) that there is a certain “empty hole” after the result of the frame difference detection. If the current binary image is directly detected, it will obtain a very complicated result, as shown in Figure 2(e). Performing the next test will result in a very complicated calculation.

In order to solve the problem of the frame difference method, we had improved two parts: first, the expansion operation of the binary image can fill the generated holes, as shown in Figure 2(g), and it can be seen that the result is improved:

(3) $$\begin{array}{c} F_{\text{d}}\left(x,y\right)=\left\{\begin{array}{c} \text{max }F_{t}\left(x+x{}^{\prime },y+y{}^{\prime }\right)\\ \left(x{}^{\prime },y{}^{\prime }\right):\text{element}\left(x{}^{\prime },y{}^{\prime }\right)\neq 0 \end{array}\right\}, \end{array}$$

where (*x*′, *y*′) denotes the kernel used, the expansion is the operation of finding the local maximum, the image is convolved with the kernel, the maximum value of the pixel of the region covered by the kernel is calculated, and the maximum value is assigned to the pixel in the image. This method can fill holes in the image. *F*_*d*_(*x*, *y*) is the result of expansion, as shown in Figure 2(f).

As can be seen from Figure 2(f), it can be improved; for example, the rectangles are very close to each other and can be combined. Therefore, a distance optimization method based on coordinate quadrant is proposed. The steps are as follows:

1. Make a two-dimensional coordinate system with the top left corner of one of the rectangles as the origin, and the quadrant of the top left corner of another rectangle is determined.
2. Take a different point on the rectangle for comparison according to the different quadrants.
3. Perform vector subtraction on the two points taken.
4. Calculate the distance between the two rectangles by the determined subtracted vector, and the formula is as follows, where (*x*_1_, *y*_1_) is the coordinate point of the first rectangle and (*x*_2_, *y*_2_) is the coordinate point closest to the first rectangle:

(4) $$\begin{array}{c} d=\sqrt{{\left(x_{1}-x_{2}\right)}^{2}+{\left(y_{1}-y_{2}\right)}^{2}}. \end{array}$$

In order to reduce the computational complexity and set a certain distance, the merging is performed, and if it is larger than this, no processing is performed whenever the distance between the two rectangles is smaller than this. Our distance optimization results for the expanded binary image are shown in Figure 2(h). It can be seen that our method is reasonable and performs very well.

**Figure 3:**
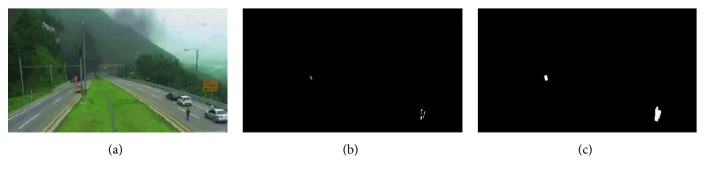
Using the frame difference method to obtain the area of interest, but nonfire pixels are still detected.

After completing the aforementioned stages, the similar areas are merged, and the coordinates of the upper left and lower right corners of the area are delivered to the color detection to complete the motion detection module. Frame difference detection is used, and a typical detection result is shown in Figure 3. It shows the result of the motion detection process.  Figure 3(a) shows the original image of a surveillance camera.  Figure 3(b) is the result of the frame difference, the actual moving pixels has interfered, and Figure 3(c) is the final motion detection result after the optimization process. There are fire pixels, but there are nonfire pixels in the lower right corner.

### 3.3. Color Detection Based on RGB and HSI Color Models

**Figure 4:**
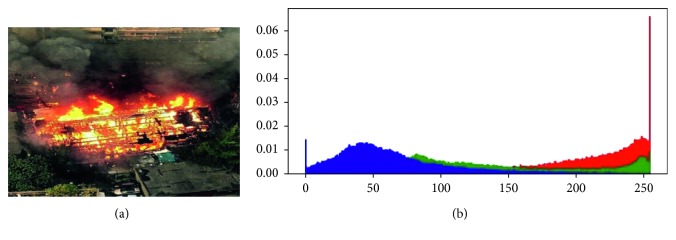
A typical flame in the RGB color model.

The most obvious feature of the static flame image is the color. According to the RGB color histogram of the flame image, the red channel value of the flame is greater than the green channel value, and the green channel value is greater than the blue channel. According to the flame area histogram, the red channel exists above a fixed value, and each pixel value in the red channel is greater than this value.  Figure 4 shows the correspondence between the flame area and the RGB color model. Blue indicates the value of the fire pixel in the blue channel, green indicates the value of the fire pixel in the green channel, and red indicates the value of the fire pixel in the red channel. The *x*-axis represents the size of the pixel. The value range is (0–255). The *y*-axis shows the frequency.

The RGB model is more appropriate for color, but it is not very suitable for human interpretation. In general, the RGB model is more suitable for color image generation, while the HSI model is suitable for image description [35]. Given the relationship between the saturation S component in the HSI model and the *R* component in the RGB model, the decision function is updated to make the color detection more accurate. The RGB model and HSI model decision functions are

(5) $$\begin{array}{c} \text{condition }1:R>\tau ,\\ \text{condition }2:R>G>B\text{,}\\ \text{condition }3:S>0.2,\\ \text{condition }4:\text{motion rect,}\\ F_{n+1}=\left\{\begin{array}{c} \text{if condition }1\text{ and condition }2\text{ and condition }3\text{ and condition }4\\ \left(\text{candidates-fire-pixel}\right)\\ \text{else }\left(\text{nonfire-pixel}\right) \end{array}\right\}. \end{array}$$

Among these, in condition 1, *R* is the pixel value of the *R* channel in the RGB image, and *τ* is the fixed threshold of the flame pixel in the *R* channel. Generally, we set it to 150. Condition 2 indicates that the *R* channel pixel value is larger than the *G* channel pixel value in the RGB image and the *G* channel pixel value is larger than the *B* channel pixel value. Condition 3 indicates that after converting the RGB model to the HSI model, the corresponding saturation value is greater than 0.2.

**Figure 5:**
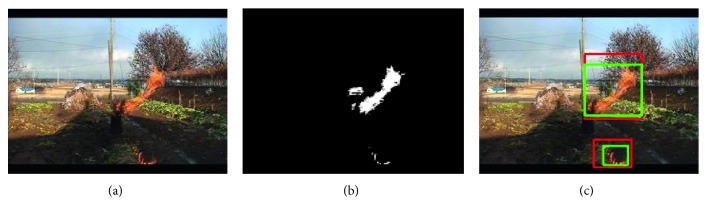
Comparison of flame color detection results and motion detection results. (b) A binary image after color detection. The red box in (c) represents the area after motion detection, and the green box represents the area after color detection. After color detection, the obtained area is more accurate.

The flame images taken by ordinary cameras are not rich in color, so we relaxed the color requirements in the first three steps. It is ensured that the fire image is not erroneously screened during the fire preprocessing stage, so images taken with ordinary cameras can also be effective. The final step gives the entrance verification process. Condition 4 indicates that the result of the previous process is used as a color detection input image. In the motion detection phase, we finally calculated the position of the rectangle where the motion pixels are located. Due to the dynamic nature of the flame, the first step is to remove nonfire pixels and retain fire pixels and the candidate for fire pixels so that we can perform the color detection of the fire based on the range of motion provided by the motion detection. Since it is necessary to detect each pixel of three channels of RGB in the color detection stage, there are a large number of calculation problems in the traditional color detection method when the image quality is high, such as 1280 ∗ 720 resolution. We proposed a preprocessing stage that achieved a significant increase in computational speed without reducing accuracy. It will be explained in the experimental results section. Finally, in the color detection stage, we counted the number of pixels in each frame of the flame and the centroid coordinates and other data, which are used to extract the advanced features of the flame in the next stage.  Figure 5 shows the color detection processed, and Figure 5(b) shows the flame pixel results obtained by color detection. In order to ensure that the flame pixels are not removed during the preprocessing stage, we relax the color detection conditions so that some nonflame pixels may be generated. Therefore, further verification is needed. As shown in Figure 5(c), we compared the results of color detection and motion detection. The candidate flame area can be reduced to improve the accuracy. At the same time, we count the flame rectangular centroid after the preprocessing stage, which is convenient for the detection of the flame space variability in the next stage. The centroid formula is shown in the following equation:

(6) $$\begin{array}{c} \left(x_{\text{c}},y_{\text{c}}\right)=\left(\frac{2\;*\text{ rect}.x+\text{rect}.\text{width}}{2},\frac{2\;*\text{ rect}.y+\text{rect}.\text{heigh}}{2}\right), \end{array}$$

where (*x*_c_, *y*_c_) is the coordinate of the centroid, rect is the rectangle holding the flame pixel, rect.*x* is the rectangle *X* coordinate value, rect.*y* is the value of the rectangle *Y* coordinate, rect.width is the width of the rectangle, and rect.heigh is the height of the rectangle.

**Figure 6:**
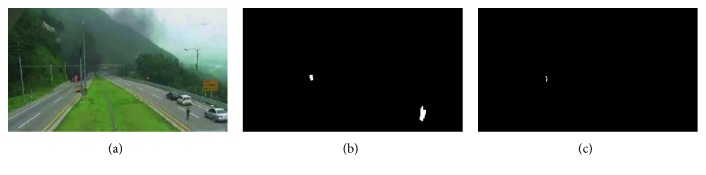
RGB color detection results. After the color model is processed, the person moving in the lower right corner is deleted, and the interfering motion pixels are deleted.

After performing motion detection on the flame image, we can obtain a motion pixel image through motion detection. Therefore, in the first step, the nonmoving objects can be removed, but some nonflame pixels that are moving may remain. After color detection, the nonflame pixels are removed, as can be seen in Figure 6.

## 4. Verification of Fire with Support Vector Machine

After the preliminary screening in Section 3, we have removed nonmoving objects and nonfire objects, but there are still a large number of nonfire pixels that constitute fire-colored moving objects, such as red moving vehicles or people wore red clothes. Thus, this paper used the flame shape change, area change, flame spatial variability, and support vector machine for verification.

### 4.1. Flame Boundary Disorder Detection

**Figure 7:**
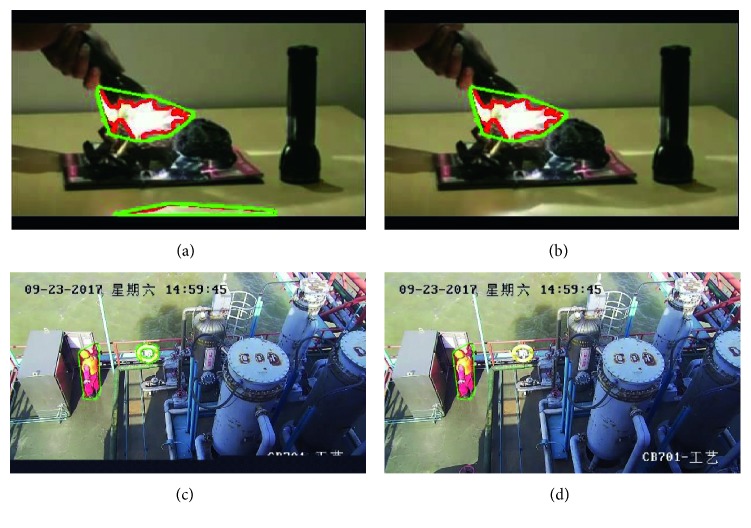
Detection results based on the convex hull.

The first feature used is the shape of the flame. The airflow and combustion properties will lead to a continuous change in the flame shape, and we used this feature to distinguish other moving objects and the real flame. We used convex hulls to calculate the disorder of the fire zone boundary. The convex hull is a set of points on a given two-dimensional plane, and the convex polygon formed by connecting the outermost points. The convex hull can contain all points in a given point set. The formula for the convex hull is as follows:

(7) $$\begin{array}{c} r=\frac{R_{\text{ch}}}{R_{\text{p}}}, \end{array}$$

(8) $$\begin{array}{c} R_{\text{p}}=\overset{n}{\underset{i=0}{\sum }}\sqrt{{\left(x_{i}-x_{i-1}\right)}^{2}+{\left(y_{j}-y_{j-1}\right)}^{2}}, \end{array}$$

where *R*_ch_ is the perimeter of the convex hull and *R*_p_ is the perimeter of the fire boundary. The perimeter of the convex hull computation uses the fast convex hull algorithm proposed by Barber et al. [36]. *r* is the ratio of the contour of the object to the convex hull of the object, and the range is (1–0). In general, the value of *r* is closer to zero, the shape of the object has obvious disorder. The closer the *r* gets to one, the regular the shape of the object is. If the value of *r* is one, it indicates that there is no flame in this image. *R*_p_ is calculated by using formula (8); (*x*_*i*_, *y*_*j*_) and (*x*_*i*−1_, *y*_*j*−1_) are the coordinates of adjacent pixels on the boundary of the connected region. The experimental results of the flame convex hull test are shown in Figure 7.  Figure 7(a) shows the original test results, and Figure 7(b) shows the results of setting a certain threshold value. The object of the boundary rule is removed. The comparison between the pictures proves that the method we use is effective.

Figure 7 is the results of convex hull detection. Among them, the red outline in the image represents the perimeter of the flame area, and the green outline represents the perimeter of the convex hull. The special characteristics of the complex object are well expressed by the convex hull. As can be seen from Figure 7(b), due to the disordered external shape of the flame, nonfire areas in Figure 7(b) is removed. Due to the flow of water and the change of the architectural shadow caused by sunlight, the nonmoving objects (lifebuoys) meet the pre-treatment conditions, as shown in Figure 7(c). The use of convex hull detection can eliminate the interference, but it is not enough to use only the convex hull because the staff with open arms satisfies the convex hull detection condition, as shown in Figure 7(d), so we need to combine with other features to improve the recognition accuracy.

### 4.2. Flame Area Change Detection

**Figure 8:**
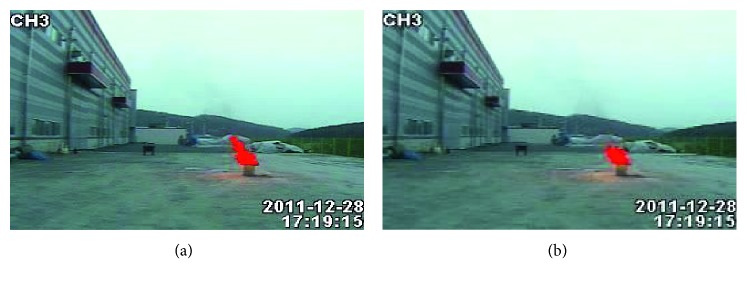
The number of fire pixels in adjacent frames. There are 1,784 fire pixels in (a) and 1,465 in (b).

According to the dynamic and disorder of the flame, the flame is always changing, so the number of flame pixels in each frame image containing the flame is constantly changing. We combined the detection results with the fire preprocessing stage, counted the candidate flame pixels of each frame image, and compared them with the next frame image. A representative example of the upper and lower frame images for flame detection is shown in Figure 8. The detection formula is as follows:

(9) $$\begin{array}{c} R_{v}=\frac{\left|S_{n+1}-S_{n}\right|}{S_{n+1}}, \end{array}$$

(10) $$\begin{array}{c} S_{n}=\underset{\left(x,y\right)\in \Omega_{i}}{\sum }f\left(x,y\right). \end{array}$$

In formula (9), *S*_*n*+1_ is the number of detected flame pixels in the current frame and *S*_*n*_ is the number of detected flame pixels in the previous frame. *R*_*v*_ is the ratio of the flame pixel difference between the two frames in the current image, which ranges from 0–1. If the value of *R*_*v*_ tends to zero, the change floats small. If the value of *R*_*v*_ tends to one, it means that the variation is large. At the same time, if the value of *R*_*v*_ is zero, the current image does not satisfy the flame property. In formula (10), *S*_*n*_ is the number of detected flame pixels in the previous frame and *Ω*_*i*_ is a connected region that needs to be measured. *f*(*x*, *y*) is the binary image pixel value.

### 4.3. Detection Algorithm Based on Spatiotemporal Relation of Flame

**Figure 9:**
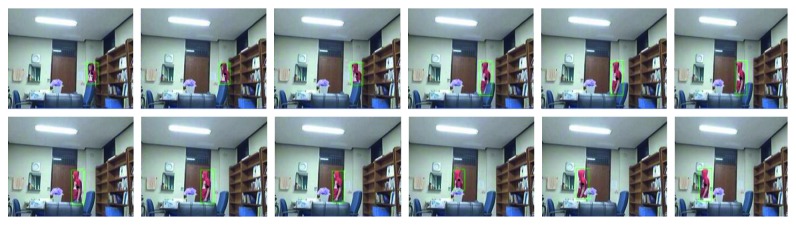
The change of the centroid of the motion and fire-color object. The green outline represents the candidate flame area after fire preprocessing (in this section, the algorithm is not used), and the black point in the green outline is the centroid of the area.

**Figure 10:**
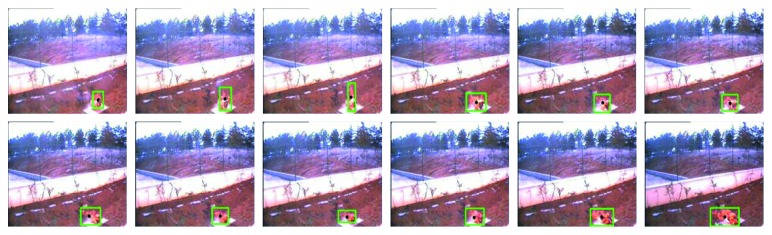
The change in the flame centroid over time.

In the above analysis, the flame is usually dynamic, and the shape is always changing. However, from the image sequence, the flames in the continuous image have a certain degree of similarity, and the flame changes have a certain range, as shown in Figures 9 and 10.  Figure 9 shows the change of the centroid of the moving object that has completed the fire preprocessing stage; the range of centroids of nonflame objects varies widely and does not have a certain range.  Figure 10 shows the change in the flame centroid over time, and the flame centroids of 12 frame images have certain similarities. The results show that the flame has a relatively stable spatial centroid in continuous time. Based on the above properties, we added the concept of time to the extraction of flame features.

**Figure 11:**
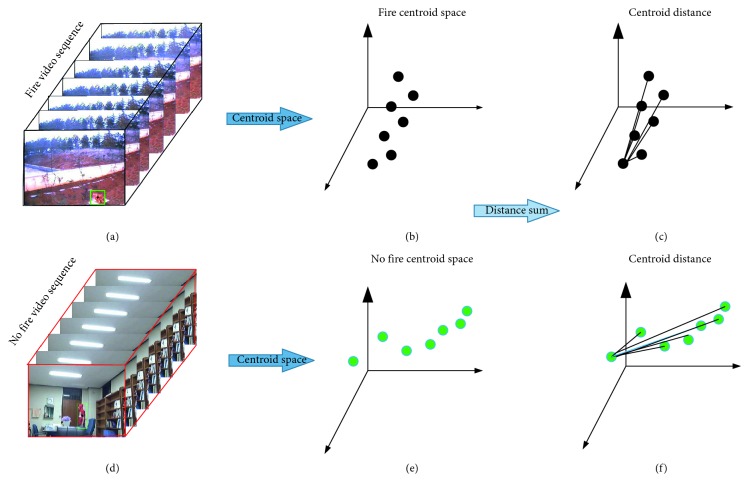
Algorithm structure.

In the study of fire detection methods, we usually use single or two frames of continuous images for research. According to the above properties, we add a time vector to the conventional detection method to assist the judgment. An algorithm for the relative stability of flame centroid based on spatiotemporal relation was proposed, as shown in Figure 11. We use the coordinate axis for representation. The *X*-axis and *Y*-axis represent a single image, and the *Z*-axis represents the time vector. In this way, we can extract the spatial centroid position change features by using multiple continuous images. The centroid formula is shown in (6).

In the preprocessing module, considering that there may be two or more fires in a picture, the centroid of the flame should be classified according to the location. For example, in Figure 2, there are two fires, so we need to consider grouping according to the position of the centroid, instead of the default image with only one centroid. We created multiple spaces to categorize and save the centroids of the video images. In the centroid preservation process, on the one hand, in the motion detection phase, we merged the rectangles of a certain distance, the distance between the rectangles is not too close, and each centroid is in the center of the rectangle, so the rectangle has a larger range with respect to the centroid. On the other hand, the flame has a certain range of variation in the continuous frame. According to the above rules, the centroid changes of the continuous frame are not very large; we used the strong correlation feature of the upper and lower frames to group multitargets situations. For example, there are two fires in the initial state of an image, and there are two centroids in the detection of the current frame image. The last added centroid in the array is taken out (the centroid of the previous frame), the shortest distance between the centroid and the array is found, and the shortest centroid is put into the corresponding array. Therefore, the grouping process of the flame centroid is completed.

After completing the centroid classification process, we obtained a series of data. According to our proposed flame spatial variability, we extracted the flame centroid data of *N* frames to judge, and the value of *N* is related to the number of frames per second of video. The distance between the centroid of the current image and the centroid of other images is, respectively, determined, and the sum of the distances is calculated. The centroid detection formula is shown in (11), the centroid distance of the 160 frames nonfire image (Figure 9) was calculated to be 16586, and the real fire (Figure 10) has a centroid distance result of 1209. Based on the difference between the two results, the effectiveness of the proposed method is proven:

(11) $$\begin{array}{c} d_{\text{sum}}=\overset{N}{\underset{i=1}{\sum }}\sqrt{{\left(x_{\text{c}}-x_{i}\right)}^{2}+{\left(y_{\text{c}}-y_{j}\right)}^{2}},\\ N=s\;*\text{ FPS}, \end{array}$$

where *d*_sum_ is the sum of the centroid change distances of the flame space. If the value of *d*_sum_ is large, it indicates that the motion of the object is not within a certain range. If the value of *d*_sum_ is small, it indicates that the motion of the object is within a certain range. (*x*_c_, *y*_c_) is the current centroid position, and (*x*_*i*_, *y*_*j*_) is the front centroid position. *N* is the total number of video frames over a period of time. FPS is video frames per second, and *s* is the time used.

### 4.4. Final Verification of Candidate Fire Image Based on Support Vector Machine

Although many nonflame images were removed in the fire preprocessing stage, the algorithm used in the above single flame feature may have the following problems. When man dressing in clothes which looks like fire and stretch his arms, it displays boundary disorder just like fire, but they are different. When man dressing in clothes which looks like fire does a squat down, it displays an area of change just like fire, but they are different. When man dressing in clothes which looks like fire worked in a small area, it displays spatiotemporal relation just like fire, but they are different. The use of a single feature of the flame may lead to the above false alarms, so it is necessary to fuse the various features of the flame to reduce the false positives of the detection. The method based on the support vector machine (SVM) is used to fuse the flame features.

The support vector machine [37] is a very mature classification model. It can optimize the classification effect by constructing a hyperplane. SVM had the flexibility to handle linear and nonlinear classification problems. In this paper, the support vector machine (SVM) is used to validate the candidate image.

#### 4.4.1. Extract Selected Flame Feature Values

First, we extracted the four features of the flame, which is used as input data for a support vector machine (SVM) model. We used the following four flame features: the shape change feature value of flame *r*, the flame pixel number variation feature value *R*_*v*_, the flame space centroid change feature value *d*_sum_, and feature (RGB color values, saturation values, and gray values) obtained during the fire preprocessing stage. The above eight eigenvalues (*r*, *R*_*v*_, *d*_sum_, *R*, *G*, *B*, saturation values, and gray values) are normalized as input data of support vector machines. The support vector machine classifier needs to estimate the features and labels of each category. Therefore, statistical “training” based on given data sets is needed to determine the decision function that separates the categories. The test results of the four features are taken as the data part of the data set, and the data are tagged. Then, the sample data set production for the SVM is finalized.

#### 4.4.2. Kernel Functions for the Support Vector Machine

There are three kernel functions in consideration: a polynomial kernel function, a radial basis kernel function (RBF), and a sigmoid kernel function. Among them, the polynomial kernel function can be defined by the user according to his own needs and is more flexible, but it has more required parameters. The radial basis kernel function is similar to the Gaussian distribution and is also called the Gaussian kernel function. Compared to polynomial kernel functions, radial basis kernel functions required fewer parameters and can save training time. It can map the original features in any number of dimensions and had good resilience to noise. This kernel function has been used successfully in previous fire detection research [38]. The sigmoid kernel function is derived from neural networks and is often used as a threshold function for such networks. In this paper, the SVM based on radial basis kernel function is used, as shown in the following formula:

(12) $$\begin{array}{c} K\left(x,x_{n}\right)=\exp\left\{-\frac{{\left\|x-x_{n}\right\|}^{2}}{2\sigma^{2}}\right\}, \end{array}$$

(13) $$\begin{array}{c} \gamma =\frac{1}{2\sigma^{2}}. \end{array}$$

In formulas (12) and (13), the SVM classifier based on radial basis function kernel needs to set parameters of *C* and gamma, where gamma is the parameter of the lossless function, *C* is a penalty factor, and *σ* is the width. A 10-fold cross-validation method is operated to optimize the classifier parameters (*C* and gamma) on the training set.

## 5. Result and Discussion

### 5.1. Data Set

The experiment in this article was performed on an ordinary desktop running Windows 10, with 8 GB RAM, and a 3.4 GHz Core i7 processor. To test the performance of the inspection system, the flame dataset video sequence should contain multiple fire scene detections while including fire-colored objectives as false positives. Finally, the video sequences should be recorded at different times of the day.

**Figure 12:**
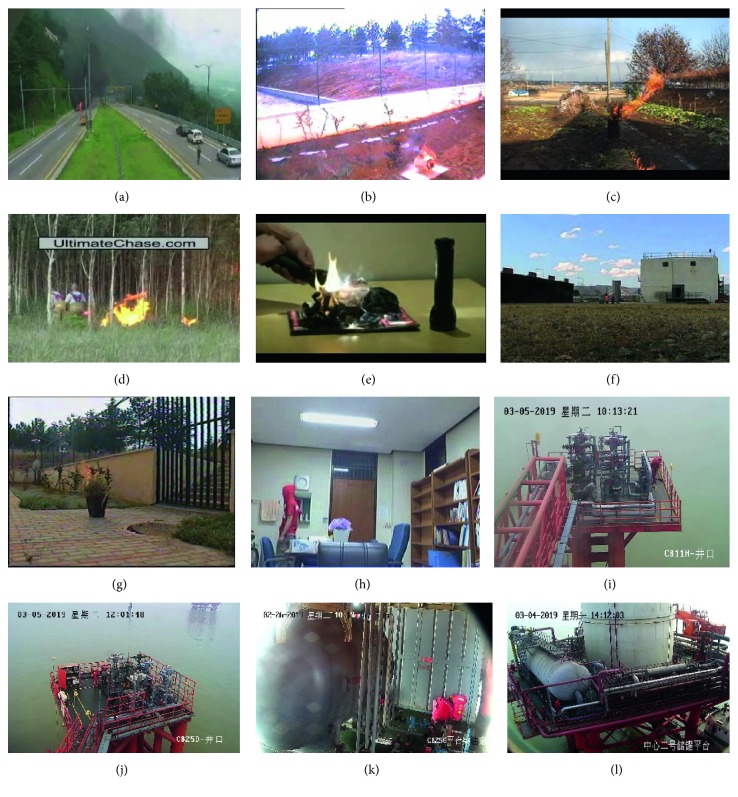
Flame video test sample. (a) Video1. (b) Video2. (c) Video3. (d) Video4. (e) Video5. (f) Video6. (g) Video7. (h) Video8. (i) Video9. (j) Video10. (k) Video11. (l) Video12.

We used a series of test videos as the fire database [35, 39–42], and added the actual scene under the program running, such as Video9 to Video12. The fire data used to support the results of this study have been stored in a repository (http://www.firedata.club). It contains various types of fires in various places. The scenes of fires include highways, outdoor, building interiors, and forests, while the nature of the fires includes highway explosions and fires in small areas, confined areas, and fields. They also include forest fires, simulated fires in indoor environments, fires near buildings and far from camera surveillance areas, and fires which can be mistaken for other moving fire-colored objects. The time of day includes daytime and dusk. Photographs were captured at different periods. This variety makes it easier to evaluate system performance under different lighting and quality conditions. We tested the proposed method using a part of the video of the flame dataset. The test video is Video1–Video12, as shown in Figure 12, and the data set video details are shown in Table 1. We divided the data set into two parts: 77% of the data was used for training (Video13–Video52) and 23% of the data was used for testing (Video1–Video12).

### 5.2. Experimental Results and Analysis

**Figure 13:**
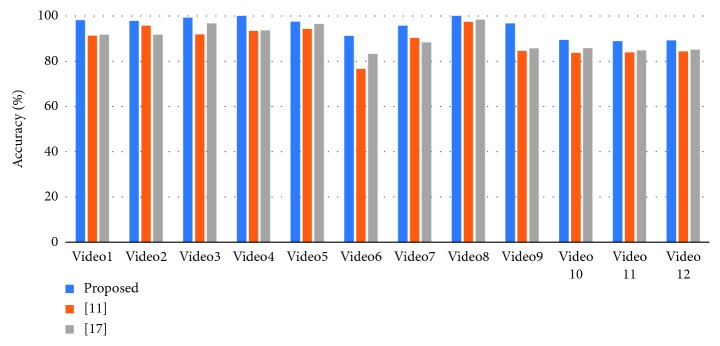
Correct frame detection rates for our method and state-of-the-art technology.

**Figure 14:**
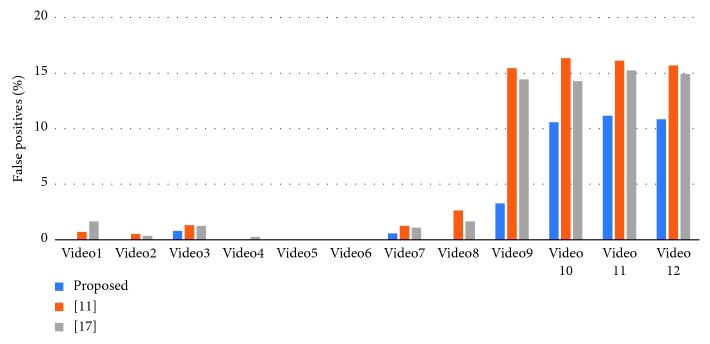
False positives rates for our method and state-of-the-art technology.

**Figure 15:**
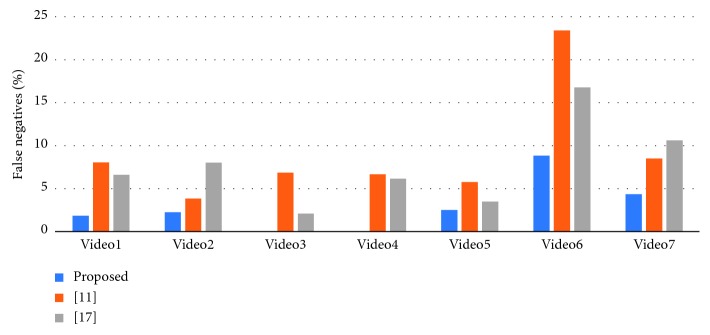
False negatives rates for our method and state-of-the-art technology.

**Figure 16:**
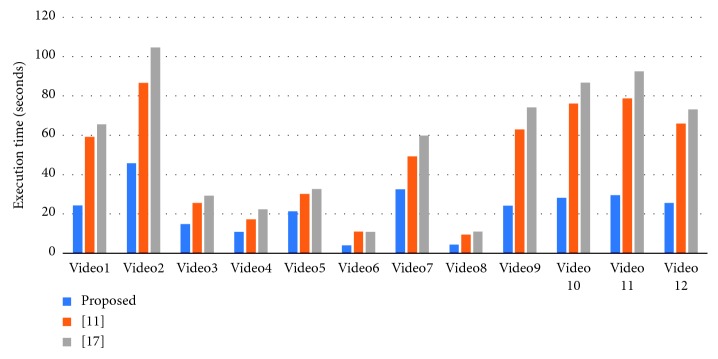
Comparison of execution time of our method and state-of-the-art technology.

To verify the effectiveness of the proposed method, we have chosen the latest method of Khan et al. [11] and Han et al. [17] for comparison experiments. Our statistical criteria are as follows. First, the program needs to correctly identify and classify fire situations. Second, we need to check whether the flame area in the fire classification image is correct.  Figure 13 shows the comparison of detection accuracy between our method and other algorithms.  Figure 14 compares the false positives rate of our method with other algorithms.  Figure 15 shows the false negatives rate of our method with other algorithms.  Figure 16 is a comparison of the execution speed of this method with state-of-the-art technology.

First, we used two evaluation metrics. In a pattern recognition task, we need to define the true positives and the false positives. We used the accuracy of regional-level flame detection to perform statistics and used IoU (intersection over union) as our evaluation metrics. The intersection of the detection result (detection result) and ground truth is divided by their union, which is the accuracy of the detection. The calculation formula is as follows:

(14) $$\begin{array}{c} \text{IoU}=\frac{\text{detection result }\cap \text{ ground truth}}{\text{detection result }\cup \text{ ground truth}}. \end{array}$$

**Figure 17:**
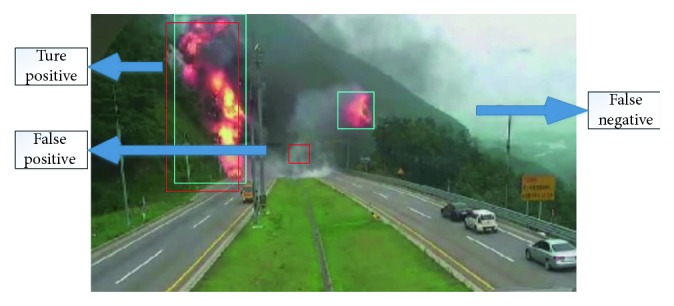
Definitions of TP, FP, and FN.

If IoU > 0.5 between the predicted box and the ground truth box, the predicted box is a “true positive (TP)”, otherwise, it is a “false positive (FP)”, and we measure “false negative (FN)” as the objects that the model has missed out (Figure 17). The predicted bounding boxes are red, and the ground truth boxes are blue.

The accuracy of the classifier is defined as

(15) $$\begin{array}{c} \text{accuracy}=\frac{\text{TP}+\text{TN}}{\text{TP}+\text{FP}+\text{TN}+\text{FN}}. \end{array}$$

We can analyze the experimental results according to Figures 13–16. As can be seen from Figure 13, the algorithm proposed in this paper has higher accuracy compared with [11] and [17].

**Figure 18:**
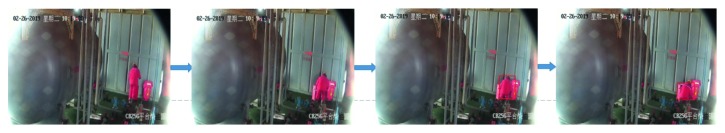
The false positive result of Video11.

Second, according to Figure 14 of false positives rates, the algorithm of [11] and [17] have a low detection false positives rate and a good performance for video of ordinary flame (Video1–Video7). However, the detection effect of the nonflame image is not very good, especially when the target and flame are very similar, and the false positives rate will be higher. The proposed algorithm performs better than the algorithms in [11] and [17] in the false positives rate. The reason for the false positive in Video7 is that there is overlap between nonflame targets and flame targets in the video. Although flame is included in the detection result, this situation does not meet our statistical standard, so it is regarded as a false positive. Our method still performs well in complex scenes (in Video9–Video12, many pillars are similar to the flames, and the staff dressing in clothes which look like fire). This is because we extracted four advanced features that are very effective in flames and combined these features to effectively reduce the occurrence of false positives and improve the robustness of the algorithm. However, our algorithms also have limitations. For example, when employees worked in complex environments, his or her body may be blocked by buildings and caused false positive, as shown in Video10 and Video12, or when employees squatted within a certain range for a short time (as shown in Video11, rapid changes occur within a second). The false positive result of Video11 is shown in Figure 18.

Third, we can see from Figure 15 that our proposed algorithm has a lower rate of false negatives. Since there was no fire in Video8–Video12, we removed this part of the data in the false negative rate statistics. At the same time, the method [11] may result in a high false negative rate due to the abandonment of a small flame. Finally, we can see from Figure 16 that the time method required for the execution of the detection is compared with the methods of [11] and [17]. According to the data in the figure (since Video9–Video12 takes a long time, we test the video according to the 700-frame standard), we find that the advantages of our method can be more prominent when detecting high-resolution video (such as Video1 and Video9–Video12). If we process long-term video (such as Video2), the gap will become obvious. We can see that the difference is not so large when detecting Video5, which may be due to the lower resolution of the video (320 ∗ 240) and the video has more motion areas, but even in this case, we can see that the method is a certain advantage.

It can be seen from Table 2 that the exclusion of any one of the features will influence the accuracy of the algorithm. Where the MC represents the motion detection and color detection of the input image, the BD represents the disorder feature of the flame boundary, the AC represents the change feature of the flame area, and ST-C represents the centroid stability feature of the flame based on spatiotemporal relation. As can be seen from Table 2, there were a large number of false positives in the detection results of MC. An advanced feature of flame combined with preprocessing results improved accuracy. Among the three advanced features, the best contribution rate of independence is the AC. We can also see that the final decision of the fusion of the four characteristics of the flame (including preprocessing features) can achieve higher accuracy and lower false positives rate; at the same time, it will increase the false negatives rate. The three features of the flame are independent of each other and have no duplicate features, so the method based on the multifeature fusion of flame obtained better accuracy.

The threshold in formula (2) is very important and is not constant for all video sequences.  Table 3 shows the influence of threshold selection on detection results. It can be seen from Table 3 that setting the threshold value to 20 in most scenes can achieve better results, but in the distant shooting of the camera, such as Video6, a small threshold value can improve the accuracy. If the camera is close, such as Video2, Video5, and Video11, a threshold of 30 can achieve better results.

The experimental results demonstrate that the accuracy of the fire detection algorithm based on multifeatures proposed in this paper is higher than that of other algorithms. At the same time, the algorithm proposed in this paper shows superior performance in minimizing false positives and false negatives. It is quicker and more stable than other algorithms. In terms of the time required for algorithm execution, the proposed method combined motion detection and color detection in the preprocessing stage, and the preprocessing results were used for the extraction of advanced flame features, so this method greatly reduces the time required by the program. The statistics from the preprocessing phase also shortened the calculation time. Therefore, this algorithm is superior to state-of-the-art technology and can truly meet real-time fire detection.

The final test results show that the correct detection rate is 95.29%, and we better combined the fire image preprocessing module to make our detection speed faster and achieved real-time detection. According to the experimental results, the fire detection algorithm based on multifeatures proposed in this paper can accurately identify fires and can identify the fire in earlier stages.

## 6. Conclusion

In this paper, a new video-based flame multifeatures fusion fire detection method was proposed. Our new approach has many advantages. First of all, our method has three key steps in the fire preprocessing stage. First, the combination of motion detection and color detection greatly improved the detection speed and reduced the redundancy calculation. Second, the motion detection was based on the improved frame difference method. Third, the fire preprocessing stage can be combined with the subsequent advanced feature extraction of the flame to reduce the calculation time. Then, the important visual features of the flame were used, such as color, motion, area change, shape change, and a novel algorithm of flame centroid stabilization based on spatiotemporal relation is proposed. Finally, the support vector machine is used to get the best result.

According to the experimental results, the correct rate of the proposed method is close to 95.29%, which proved that our proposed new method has higher accuracy and stability. However, our algorithms also have certain limitations, such as when the staff dressing in clothes which looks like fire is blocked by the equipment (as shown in Video10 and Video12), or when the staff has a pick-up action within a certain range (as shown in Video11), these conditions are in line with our proposed flame advanced features, so it may cause false positives. In the future, optimization should be carried out while trying to use more advanced methods to solve the problems faced by the actual environment. For the next step, according to temporal information, we will look for more effective ways to resolve existing false positives.

## Figures and Tables

**Table 1:** Data set video sequence.

Video sequence	Number of frames	Description	Resolution
Video1	789	Highway (outdoor)	640 ∗ 360
Video2	1201	Field (outdoor)	320 ∗ 240
Video3	402	Farm (outdoor)	320 ∗ 240
Video4	260	Forest (outdoor)	400 ∗ 256
Video5	411	Close flame (indoor)	320 ∗ 240
Video6	140	Very long distance situation (outdoor)	320 ∗ 240
Video7	708	In the presence of interference (outdoor)	320 ∗ 240
Video8	171	No fire (indoor)	320 ∗ 240
Video9	7704	No fire (outdoor)	1280 ∗ 720
Video10	41304	No fire (outdoor)	1920 ∗ 1080
Video11	1600	No fire (outdoor)	1920 ∗ 1080
Video12	6300	No fire (outdoor)	1280 ∗ 720

**Table 2:** Four features of flame contributing to the detection accuracy.

Method	Accuracy (%)	False positives (%)	False negatives (%)
MC	82.17	17.83	0
MC + BD	85.65	13.57	0.78
MC + AC	89.52	10.48	0
MC + ST-C	88.96	10.51	0.53
MC + BD + AC + ST-C	95.29	3.09	1.62

**Table 3:** Selected thresholds for all video sequences.

Thresh video	10 (%)	15 (%)	20 (%)	30 (%)	40 (%)
Video1	95.38	97.12	**98.16**	95.37	92.18
Video2	93.12	97.01	97.45	**97.83**	95.69
Video3	97.34	98.37	**99.20**	98.11	93.26
Video4	98.27	99.11	**100.00**	98.05	96.24
Video5	93.57	96.25	97.50	**97.96**	94.27
Video6	91.05	**92.21**	91.27	83.23	79.59
Video7	93.78	94.26	**95.65**	95.07	88.32
Video8	90.71	97.05	**100.00**	99.16	98.61
Video9	95.17	96.01	**96.82**	95.52	93.88
Video10	82.10	88.91	**89.43**	86.21	83.22
Video11	81.25	85.97	88.84	**89.10**	87.25
Video12	87.27	89.03	**89.14**	88.34	87.03

## Data Availability

The data used in this manuscript have been uploaded at the website http://www.firedata.club, which is completely open and free for readers and researchers.
